# Real-Time Flange Bolt Loosening Detection with Improved YOLOv8 and Robust Angle Estimation

**DOI:** 10.3390/s25196200

**Published:** 2025-10-06

**Authors:** Yingning Gao, Sizhu Zhou, Meiqiu Li

**Affiliations:** School of Mechanical Engineering, Yangtze University, Jingzhou 434023, China; gyn_gn@163.com (Y.G.); zhouszcjdx@163.com (S.Z.)

**Keywords:** bolt loosening detection, YOLOv8, MobileViT, multi-scale feature fusion, angle estimation

## Abstract

Flange bolts are vital fasteners in civil, mechanical, and aerospace structures, where preload stability directly affects overall safety. Conventional methods for bolt loosening detection often suffer from missed detections, weak feature representation, and insufficient cross-scale fusion under complex backgrounds. This paper presents an integrated detection and angle estimation framework using a lightweight deep learning detection network. A MobileViT backbone is employed to balance local texture with global context. In the spatial pyramid pooling stage, large separable convolutional kernels are combined with a channel and spatial attention mechanism to highlight discriminative features while suppressing noise. Together with content-aware upsampling and bidirectional multi-scale feature fusion, the network achieves high accuracy in detecting small and low-contrast targets while maintaining real-time performance. For angle estimation, the framework adopts an efficient training-free pipeline consisting of oriented FAST and rotated BRIEF feature detection, approximate nearest neighbor matching, and robust sample consensus fitting. This approach reliably removes false correspondences and extracts stable rotation components, maintaining success rates between 85% and 93% with an average error close to one degree, even under reflection, blur, or moderate viewpoint changes. Experimental validation demonstrates strong stability in detection and angular estimation under varying illumination and texture conditions, with a favorable balance between computational efficiency and practical applicability. This study provides a practical, intelligent, and deployable solution for bolt loosening detection, supporting the safe operation of large-scale equipment and infrastructure.

## 1. Introduction

Flange bolt connections are utilized extensively in civil, mechanical, and aerospace engineering due to their reliability and versatility. The preload stability of these components directly affects connection stiffness and structural safety. However, cyclic loads, vibrations, and environmental disturbances may gradually lead to loosening, enlarged gaps, and even catastrophic accidents [1]. Consequently, the efficient and accurate detection of bolt loosening without interrupting structural operation has become a central issue in structural health monitoring and quality management.

Conventional inspection approaches are predominantly characterized by manual visual inspections and the utilization of torque wrenches for measurement [2,3]. While these methods are straightforward, they are labor-intensive, prone to errors, and impractical for large-scale infrastructure with thousands of fastening points. Contact-based sensor methods, including strain gauges, guided waves, and acoustic emission [4,5,6,7], offer quantifiable data but encounter challenges such as high cost, intricate deployment, and unstable performance in harsh environments [8]. These limitations have prompted the development of non-contact solutions that offer efficiency, safety, and scalability.

Among non-contact techniques, remote vibration or audio-based measurements and image-based computer vision have been the focus of extensive investigation [9,10,11,12]. The former relies on excitation–response disparities, but it is highly sensitive to background noise and coupling conditions. The latter employs object detection and geometric analysis to assess bolt states. Early image-based methods relied on handcrafted features, whereas recent advancements in deep learning have enabled more reliable detection and angular estimation [13,14]. Nevertheless, contemporary visual methodologies continue to struggle with challenges in complex backgrounds, substantial illumination variations, and dense multi-bolt distributions, where balancing robustness and real-time performance remains a formidable task.

In the domain of real-time visual detection, the You Only Look Once (YOLO) series has been widely applied. For instance, YOLOv3 and YOLOv4 have been augmented with capabilities for bolt localization, numbering, and rotation monitoring [15,16,17]. However, their effectiveness is constrained by limited feature representation and insufficient cross-scale fusion. This underscores the necessity for further architectural refinement and the development of robust angle estimation pipelines to meet industrial deployment requirements. Recent work [18] reviews AI-enabled self-reconfigurable manufacturing and highlights the importance of real-time condition monitoring and edge/intelligent perception modules in industrial environments, which is closely related to vision-based monitoring tasks such as bolt loosening detection.

**Figure 1:**
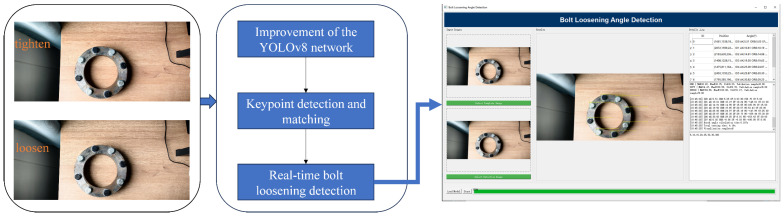
Diagram of the overall architecture of the framework.

To address these challenges, this study proposes a vision-driven automatic bolt loosening detection framework tailored for complex industrial scenes. The overall architecture of the framework is illustrated in Figure 1, which provides a high-level view of the system modules and information flow to guide the reading of subsequent sections. In this context, our objective is to develop a robust and real-time visual framework that accurately detects bolts and estimates their rotation angles under complex backgrounds and illumination, enabling scalable, non-contact loosening monitoring suitable for industrial deployment. To achieve this objective, we design a lightweight, attention-enhanced detection network balancing global context modeling and efficient inference; construct a training-free, noise-resilient angle estimation pipeline that avoids manual markers and extra annotations; integrate detection and angle estimation into an online system for multi-bolt monitoring and spatial localization on edge/industrial terminals; and conduct comparative and ablation experiments to validate robustness, accuracy, and efficiency.

The contributions can be categorized into three distinct aspects:

1. Enhanced detection network: The proposed method is built upon a lightweight YOLOv8 architecture. It integrates a Mobile Vision Transformer (MobileViT) backbone [19], a Large Separable Convolutional Kernel Attention (LSKA) module [20], and a Convolutional Block Attention Module (CBAM) [21]. These components are further combined with content-aware upsampling and Bidirectional Feature Pyramid Network with Cross-Stage Partial Units in Improved Bottlenecks (BiFPN + C2f_UIB) [22] for multi-scale fusion, thereby improving the accuracy and robustness of small-target detection.
2. Training-free angle estimation pipeline: A robust estimation process is constructed based on Oriented FAST and Rotated BRIEF (ORB) feature detection [23], Fast Library for Approximate Nearest Neighbors (FLANN) matching [24], and Random Sample Consensus (RANSAC) fitting [25]. This workflow reliably extracts stable rotational components under reflection, blur, and viewpoint deviations, without reliance on manual annotations or markers.
3. Integrated software system: The proposed system cascades detection and angle estimation results, enabling online monitoring, anomaly labeling, and spatial localization of multiple bolts. This implementation is well-suited for deployment on edge computing platforms and industrial terminals.

The remainder of this paper is organized as follows. Section 2 provides an overview of the proposed framework and the design of key modules. Section 3 introduces the experimental setup and dataset. Section 4 presents comparative and ablation studies, followed by detailed analysis. Section 5 concludes the paper and outlines directions for future research.

## 2. Methodology

### 2.1. Overview

In order to address the demand for high-precision and highly robust bolt detection in industrial settings, a deep learning framework integrating attention mechanisms with efficient feature representation has been developed. The system design prioritizes engineering applicability and comprises three core components: The proposed methodology is composed of three sequential steps. First, a deep-enhanced YOLOv8 object detection backbone is employed. Second, bolt keypoint extraction and highly robust matching are conducted. Third, intelligent determination of bolt loosening status is made. The system is capable of processing raw images containing flange bolt structures and generating estimated spatial positions and pose angles of bolts. This functionality provides reliable data support for subsequent health monitoring and automated maintenance.

**Figure 2:**
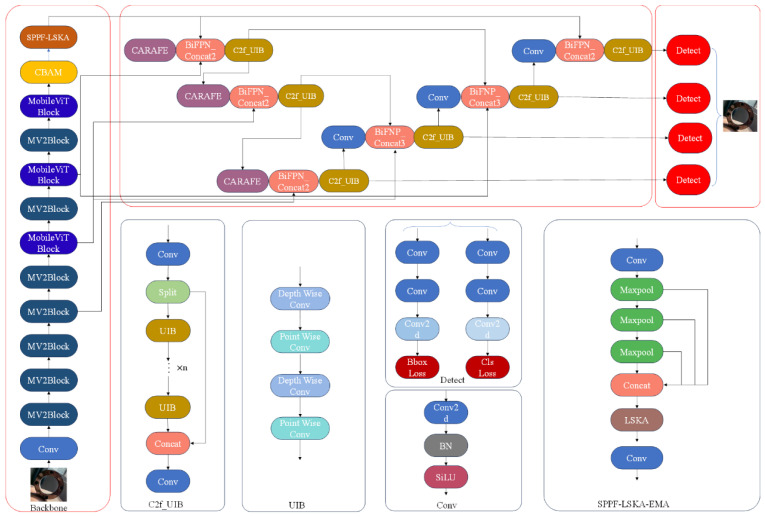
Improved YOLOv8 network structure.

In addressing the industrial challenges of large target scale variations, strong background interference, and stringent real-time constraints, this paper performs structural-level reconstruction of YOLOv8. It introduces a lightweight visual Transformer backbone to balance global modeling and efficient inference. It also designs a feature enhancement mechanism that fuses spatial–channel multidimensional attention. Furthermore, it achieves synergistic improvements in speed and accuracy through efficient feature fusion and loss constraints. The overall network architecture is illustrated in Figure 2.

### 2.2. Improved YOLOv8-Based Bolt Detection

#### 2.2.1. MobileVit Backbone Network

To provide both lightweight inference and global context modeling, this subsection introduces the MobileViT backbone, which couples convolutional layers for local edge representation with a Transformer block for long-range dependencies, followed by a fusion step for multi-scale feature integration. This high-level design ensures stronger robustness in dense and complex scenes compared to the conventional YOLOv8 backbone.

The conventional YOLOv8 architecture utilizes CSPDarknet, a technique that offers robust local feature representation capabilities but is constrained in jointly modeling global dependencies and multi-scale information. This limitation frequently results in missed detections in scenes characterized by density and complexity. To address this, the present paper adopts MobileViT as the foundation, deeply integrating lightweight convolutional networks with Vision Transformers to balance edge inference efficiency and global semantic aggregation capabilities.

**Figure 3:**
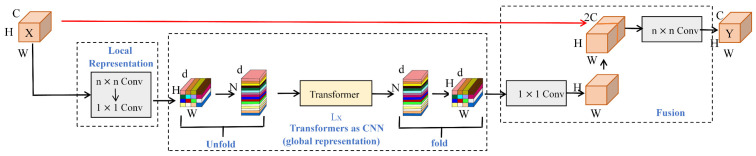
MobileVit Block structure diagram: left—Local Representation → Equation (1); middle—Unfold → Transformer → Fold → Equations (2)–(4) with token dimension d; right—Fusion → Equation (5). C/H/W denote channels/height/width; the red arrow indicates cross-module skip/cascade information flow.

**Figure 4:**
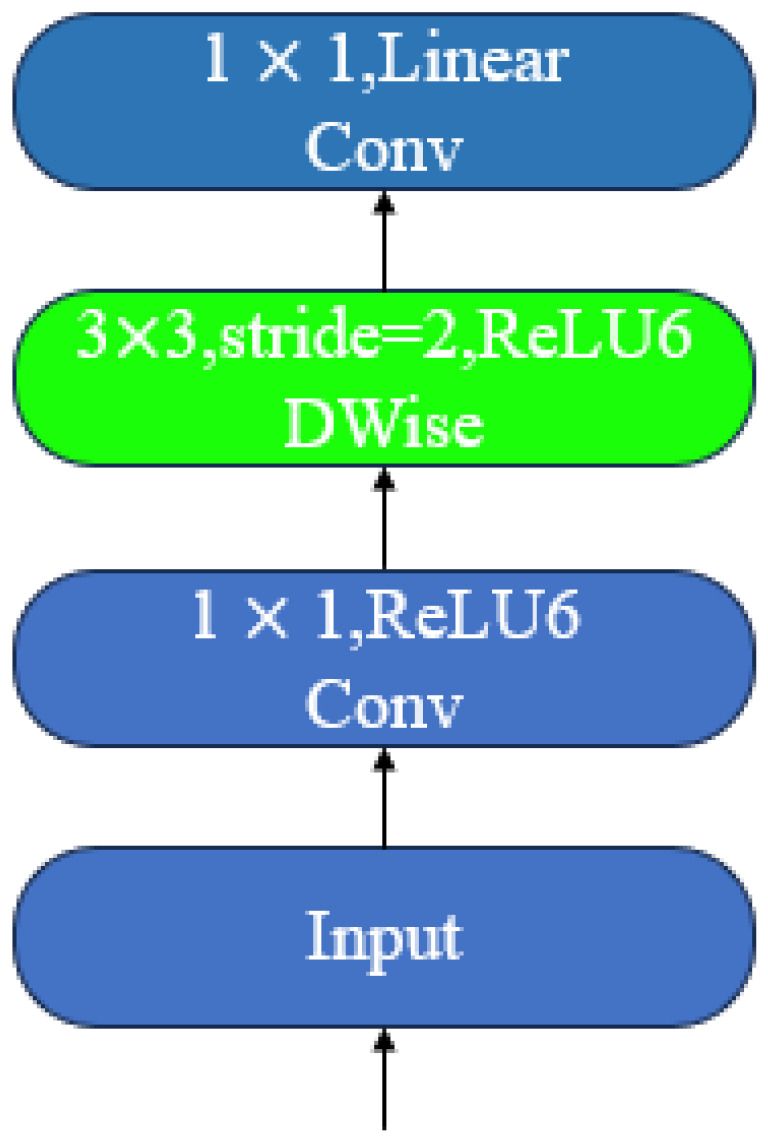
MV2Block.

The MobileViT network architecture is illustrated in Figure 3 and Figure 4. For clarity, Figure 3 aligns with Equations (1)–(5) as follows: the left block “Local Representation” corresponds to Equation (1); the middle block “Unfold → Transformer → Fold” corresponds to Equations (2)–(4), where features are projected to dimension d, unfolded into tokens, and globally modeled; the right block “Fusion” corresponds to Equation (5), where the restored feature is concatenated with the local branch and compressed by a 1 × 1 convolution. Here, C, H, W denote channels/height/width, d is the token embedding dimension, and the long red arrow indicates the cross-module skip/cascade information flow.

The core module of the system, designated as the MobileViT Block, comprises three distinct stages.

(1) Local Feature Extraction:

A 3 × 3 convolution is applied to perform preliminary modeling of the input image features $X_{in}\in R^{H\times W\times C}$, enhancing local edge and texture responses:

(1) $$F_{local}=Conv_{3\times 3}(X_{in})$$

In this context, $F_{local}$ denotes the local spatial feature, while H, W, and C represent the height, width, and number of channels of the feature map, respectively.

(2) Global Information Encoding:

The initial step in the procedure is to elevate $F_{local}$ to a d-dimensional space via a 1 × 1 convolution. Subsequently, the feature map is unfolded into multiple token sequences and fed into the Transformer Block for global self-attention modeling:

(2) $$F_{embed}=Conv_{1\times 1}(F_{local})$$

(3) $$F_{trans}=Transformer(Unfold(F_{embed}))$$

The core mechanism within the Transformer is the multi-head self-attention calculation formula, as follows:

(4) $$Attention(Q,K,V)=softmax\left(\frac{QK^{T}}{\sqrt{d_{k}}}\right)V$$

In this model, *Q*, *K*, and *V* represent the query, key, and value vectors, respectively, and $d_{k}$ denotes the dimension of the attention head.

(3) Multi-scale Fusion Output:

The Transformer output $F_{trans}$ restores spatial structure through a Fold operation, concatenates with the original local features, and then fuses the outputs via a 1 × 1 convolution to form a multi-scale contextual representation:

(5) $$F_{out}=Conv_{1\times 1}(Concat(F_{local},Fold(F_{trans}))$$

This enhancement signifies a substantial augmentation in the modeling capabilities for small objects and global dependency modeling, characterized by a reduction in parameters and accelerated inference speeds. The system’s suitability for integration into edge computing or industrial terminals is evident, as it is capable of handling highly robust detection tasks across a range of scales, complex lighting scenarios, and occlusion conditions.

#### 2.2.2. SPPF-LSKA Module

To strengthen feature aggregation across scales, this module enhances YOLOv8’s SPPF layer by embedding a large-kernel attention mechanism, thereby expanding the receptive field and capturing richer spatial–semantic dependencies without sacrificing efficiency.

In YOLOv8, SPPF utilizes fixed-size pooling operations to extract features, thereby resulting in a fixed receptive field that limits the capture of larger-scale features. Additionally, the feature fusion process exhibits an inability to extract global contextual information, resulting in an inadequate capture of spatial relationships and semantic information among objects.

The Large Convolution Kernel Attention (LKA) module integrates the advantages of both convolutional and self-attention mechanisms, including local structural information, long-range dependencies, and adaptability. This approach effectively addresses issues such as adaptive neglect in the channel dimension. Large-kernel convolution comprises three primary components: depth-wise convolution (DWC), depth-wise dilation convolution (DWDC), and 1 × 1 convolution.

The calculation formula for the LKA module is shown in Equation (6).

(6) $$LKA(x)=C^{1\times 1}(DWD\_C(DW\_C(x)))$$

In this context, x denotes the input feature map, *LKA(x)* represents the attention output feature map, $C^{1\times 1}$ indicates a 1 × 1 convolution, *DW_C* denotes a depth-wise convolution operation, and *DWD_C* signifies a depth-wise dilation convolution.

**Figure 5:**
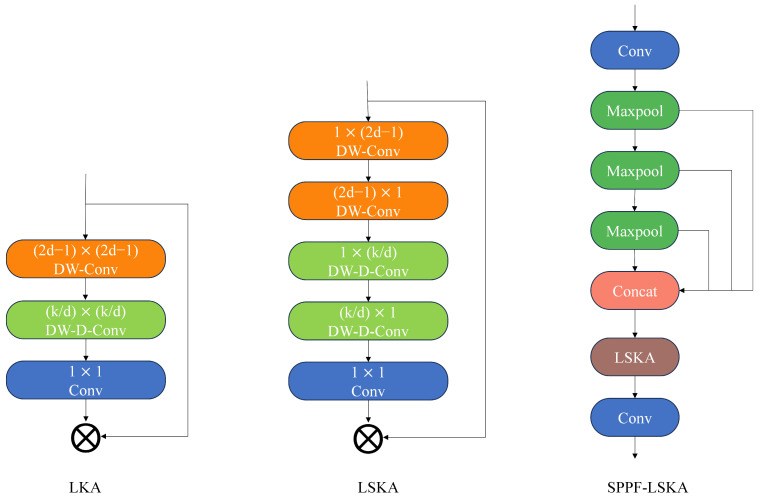
SPPF-LSKA module diagram.

LSKA decomposes the two-dimensional convolution kernels in the deep convolution layers of LKA into cascaded horizontal and vertical one-dimensional convolution kernels. In comparison to the original LKA, it has been demonstrated to achieve a substantial enhancement in performance while concurrently reducing computational complexity and memory consumption. Therefore, to enhance SPPF’s feature extraction capability for input feature maps, LSKA is incorporated into the SPPF layer, forming the SPPF-LKSA layer. The network architecture is illustrated in Figure 5.

#### 2.2.3. Content-Aware Reassembly Feature Embedding

To improve recovery of fine-grained details during upsampling, YOLOv8’s default nearest-neighbor interpolation is replaced with the CARAFE module, which leverages content-aware kernels to better preserve small-object features while maintaining lightweight computation.

**Figure 6:**
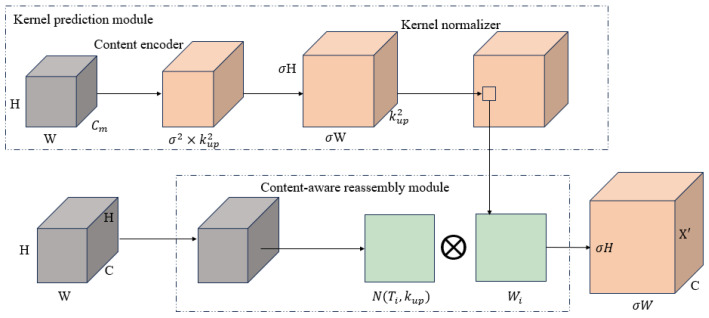
CARAFE structure diagram.

The implementation of nearest-neighbor interpolation upsampling in YOLOv8 does not consider local context, resulting in the loss of small-object features. The present study utilizes the CARAFE module for the purpose of feature upsampling, thereby achieving a substantial enhancement in semantic recovery capability, as illustrated in Figure 6.

CARAFE is an end-to-end, content-aware upscaling mechanism that achieves spatial information reconstruction through learnable kernels, demonstrating superior small-object detail recovery compared to traditional upscaling methods. The CARAFE operation is composed of two primary modules: the Kernel Prediction Module and the Content-Aware Reassembly Module.

The kernel prediction module processes input feature $X_{in}\in R^{H\times W\times C}$ through 1×1 convolutional compression channels, where the $k_{up}\times k_{up}$ convolutional kernel generates a reconstructed kernel for each pixel:

(7) $$W=softmax(Conv_{k_{up}\times k_{up}}(Conv_{1\times 1}(X_{in})))$$

In this context, *W* denotes the adaptive reconstruction kernel for each spatial position, and *k_up_* signifies the kernel size.

In the content reconstruction module, for each output position, the weighted sum of the surrounding $k_{up}\times k_{up}$ region pixels is calculated.

(8) $$X_{i,j}^{\prime }=\sum_{\left(u,v)\in N(i,j\right)}W_{i,j}(u,v)\cdot X_{in}(u,v)$$

In this context, N(i,j) denotes the $k_{up}\times k_{up}$ neighborhood centered at (i,j) and ${X^{\prime }}_{i,j}$ represents the upsampled output pixel.

The CARAFE upsampling operation has the capacity to generate distinct upsampling kernels for different features within the input image. This property enables greater focus on the distribution of features across the global feature map of the input sample. The substitution of the upsampling module in YOLOv8 with CARAFE enhances the capacity to identify salient features in the target image during upsampling without increasing the computational load of additional parameter kernels. This enhancement contributes to the refinement of extraction capabilities concerning bolt target features.

#### 2.2.4. CBAM

In order to enhance feature discriminability while maintaining lightweight properties, this paper introduces the Convolutional Block Attention Module (CBAM) into the network. CBAM is a lightweight attention mechanism comprising two submodules. It is imperative to direct attention towards both channel and spatial aspects. It facilitates explicit modeling of input features on a per-channel and per-position basis without a substantial increase in parameter count or computational overhead.

For the input feature map, $F\in R^{C\times H\times W}$, CBAM first models global semantics through channel attention:

The application of global average pooling and global max pooling to compress the feature map yields two description vectors.

These values are subsequently aggregated following the transmission through a shared multi-layer perceptron (MLP) and are then activated via Sigmoid to derive the channel attention weights.

The computation of the channel attention weights is then performed as follows:

(9) $$M_{c}(F)=\sigma (MLP(AvgPool(F))+MLP(MaxPool(F)))$$

The subsequent step involves multiplying the original features by the same number to obtain the channel-enhanced features.

(10) $$F^{\prime }=M_{c}(F)⊗F$$

Subsequently, CBAM employs a spatial dimension in its modeling of $F^{\prime }$.

The model performs two types of pooling operations: global average and max pooling. These operations are performed along the channel dimension. The results of the pooling operations are then concatenated. Subsequently, a convolution operation is applied to generate spatial attention weights.

(11) $$M_{s}(F^{\prime })=\sigma (f^{7\times 7}([AvgPool(F^{\prime });MaxPool(F^{\prime })]))$$

The following spatial enhancement features were obtained:

(12) $$F\prime \prime =M_{s}(F^{\prime })⊗F^{\prime }$$

The present study posits that CBAM should precede SPPF-LSKA (situated at the convergence of the trunk and neck) principally due to the sensitivity of SPPF’s multi-scale pooling and LSKA’s large-kernel convolution to local contrast and background noise in input features. By implementing joint channel–spatial weighting prior to these operations, CBAM enhances inter-class discriminative power while suppressing redundant responses. This facilitates subsequent pyramid pooling and large-kernel convolution, allowing them to operate on more discriminative features. Consequently, microstructures such as bolt edges, holes, and engravings are more effectively highlighted. BiFPN employs a learnable weighting scheme to achieve robust discriminative fusion of cross-scale information. Subsequent incorporation of a lightweight attention module may result in functional overlap with the weighting process. Conversely, the placement of CBAM upstream provides a form of “pre-enhancement,” delivering higher signal-to-noise ratio features to SPPF-LSKA while avoiding conflicts with BiFPN’s fusion weights.

#### 2.2.5. Multi-Scale Fusion Structure with BiFPN and C2f_UIB

To further improve detection under complex backgrounds and scale variations, the neck integrates BiFPN (Bidirectional Feature Pyramid Network) for efficient bidirectional feature fusion and C2f_UIB for lightweight yet expressive channel modeling. Together, they provide stronger small-target recognition and stable gradient propagation without excessive computational cost.

In the context of deep detection networks, the primary function of the neck structure is to facilitate efficient interaction and integration of cross-scale features. This enables the detection head to recognize targets of varying sizes. This paper employs a combination of BiFPN and an enhanced C2f_UIB module within the neck section of YOLOv8 to further enhance the detection capabilities for small targets and complex backgrounds.

BiFPN is an architecture of a feature pyramid that functions in both directions. In comparison with conventional PAN-FPN, the primary enhancement of this approach lies in the concurrent introduction of top-down and bottom-up information flows. This ensures a more comprehensive interaction between feature layers of varying resolutions. BiFPN employs a learnable weighted fusion strategy for the output feature $P_{i}^{out}$ of layer i.

(13) $$P_{i}^{out}=\frac{\sum_{j\in \Omega (i)}w_{ij}\cdot {\hat{P}}_{j}}{\sum_{j\in \Omega (i)}w_{ij}+\varepsilon }$$

In this context, Ω(i) is equivalent to the set of connected input features at layer i. ${\hat{P}}_{j}$ signifies the input features subsequent to channel alignment and scale transformation. $w_{ij}$ denotes the learnable fusion weight parameter. ε represents the stabilizing term, which is employed to avert zero denominators. Prior to the integration of these features, it is imperative to ensure the alignment of scales.

(14) $${\hat{P}}_{j}=R(Conv_{1\times 1}(P_{j}))$$

In this context, R(⋅) is used to denote upsampling or downsampling operations, while $Conv_{1\times 1}$ is employed for channel dimension mapping. This mechanism ensures the equivalence and adaptability of multi-scale features during information flow, thereby enabling the fused features to achieve a better balance between global and local receptive fields.

**Figure 7:**
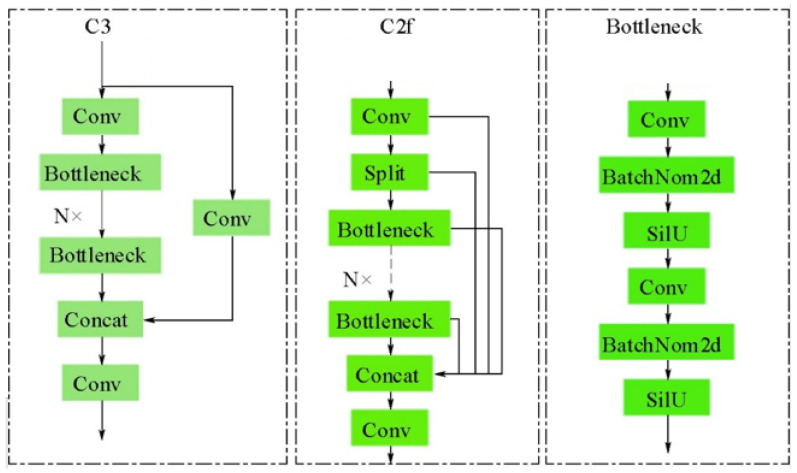
C2f structure diagram.

In order to enhance the fusion capability of BiFPN while avoiding redundant computations, this paper replaces the traditional C2f module with an improved C2f_UIB (C2f with Unified Information Bottleneck). The C2f (CSP Bottleneck with 2 convolutions) module is a lightweight structure proposed based on the C3 module. By replacing one branch’s convolutional structure with a residual structure and removing the shortcut in the C3 module, C2f significantly enriches the gradient flow. This results in C2f exhibiting a reduced weight compared to C3. Schematic representations of the C3 and C2f structures are depicted in Figure 7.

C2f achieves a balance between lightweight architecture and feature representation through its branch convolution and gradient flow separation mechanism. However, its residual bottleneck structure exhibits limitations in expressing channel dependencies and cross-layer feature reuse.

C2f_UIB builds upon this by incorporating the Unified Inverted Bottleneck (UIB) concept from MobileNetV4, replacing the original C2f module’s Bottleneck with a lightweight bottleneck structure composed of ExtraDW (Deep Convolution + Pointwise Convolution Pair). The computational process of C2f_UIB for input features $X\in R^{H\times W\times C}$ is as follows:

Initially, channel compression is to be executed via 1×1 convolution.

(15) $$F_{red}=Conv_{1\times 1}(X)$$

(16) $$F_{red}\in R^{H\times W\times \beta C}$$

In this model, β∈(0,1) represents the bottleneck compression ratio. Subsequently, a combination of deep convolutions and pointwise convolutions is employed to capture local spatial dependencies and cross-channel interactions.

(17) $$F_{dw}=DWConv(F_{red})$$

(18) $$F_{pw}=PWConv(F_{dw})$$

Subsequently, the compressed features are to be concatenated with the input features along the channel dimension.

(19) $$F_{cat}=Concat(F_{pw},X)$$

The number of channels was restored to its original state through the implementation of 1×1 convolutions, accompanied by the introduction of residual connections with the objective of mitigating gradient vanishing.

(20) $$Z=Conv_{1\times 1}(F_{cat})+X$$

**Figure 8:**
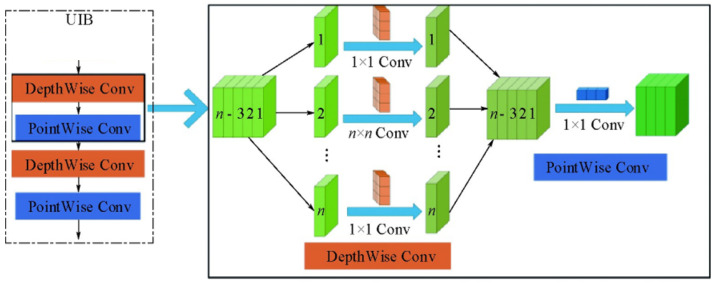
UIB structure diagram.

**Figure 9:**
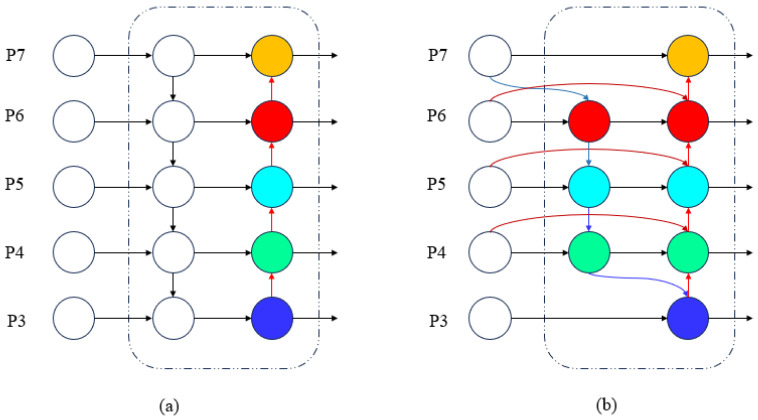
BiFPN network structure. (**a**) illustrates the conventional PAN-FPN architecture, which propagates multi-scale features through sequential top-down and bottom-up pathways. (**b**) depicts the proposed BiFPN architecture, which enhances information interaction by introducing bidirectional feature fusion and a learnable weighted aggregation mechanism, thereby achieving a more efficient and balanced multi-scale representation.

The final output, designated as Z, has been observed to preserve the global semantic information of the original features while enhancing structural details through UIB’s local modeling. This has been shown to result in a significant improvement in gradient flow and small-object discrimination capabilities. The schematic diagrams of UIB and BiFPN are shown in Figure 8 and Figure 9, respectively.

BiFPN facilitates comprehensive interaction between features of different resolutions through bidirectional paths and learnable weights, while C2f_UIB efficiently compresses and expands channel features within each fusion unit, balancing lightweight processing with expressive power. When combined, these elements ensure stable cross-level feature propagation and further enhance the fusion of local and global information.

### 2.3. Keypoint Detection and Matching

In order to achieve stable estimation of bolt rotation angles under complex conditions, such as weak textures, uneven lighting, and scale variations, this paper employs the Oriented FAST and Rotated BRIEF (ORB) technique for keypoint detection and binary description. When employed in conjunction with FLANN (Fast Localized Nearest Neighbors based on LSH), it facilitates efficient matching. Subsequently, a robust RANSAC-based estimation of the 2D affine model is performed, with the rotation angle ultimately extracted from the affine matrix. This workflow achieves a balance between real-time performance and robustness, while minimizing parameter overhead. Consequently, it is well-suited for deployment with lightweight detectors.

#### 2.3.1. ORB Keypoint Detection and Descriptor Extraction

At this stage, keypoints are identified and described to provide rotation-invariant features for subsequent matching.

(a) Multi-scale Pyramid and FAST Corners. The FAST detection process is to be executed in scale space ${\left\{I^{\left(l\right)}\right\}}_{l=0}^{L-1}$, with $I^{\left(l\right)}(x,y)=I(\frac{x}{s^{l}},\frac{y}{s^{l}})$ and s>1 denoting scale strides. For each pixel *p*, let $C(p)={\left\{qk\right\}}_{k=1}^{16}$ denote the set of Bresenham circular pixels centered at *p* with radius 3, thresholding at *T*. If a continuous arc segment of length at least *n* exists such that

(21) $$\left|\left\{q\in C(p)\;I(q)\geq I(p)+T\right\}\right|\geq n$$

(22) $$\left|\left\{q\in C(p)\;I(q)\leq I(p)-T\right\}\right|\geq n$$

Consequently, it is determined that *p* is a corner point. The Harris response is employed to suppress false responses and sort candidate points.

(23) $$R=det(M)-k\cdot tr{\left(M\right)}^{2},M=[\begin{array}{cc} \sum I_{x}^{2} & \sum I_{x}I_{y}\\ \sum I_{x}I_{y} & \sum I_{y}^{2} \end{array}]$$

where $I_{x},I_{y}$ represents the image gradient, k∈[0.04,0.06]

(b) Directional Assignment The intensity moment within the neighborhood Ω(p), centered at vertex *p* with radius r, must be calculated.

(24) $$m_{pq}=\sum_{\left(x,y)\in \Omega (p\right)}x^{p}y^{q}I(x,y),(x_{c},y_{c})=(\begin{array}{cc} m_{10} & m_{01}\\ m_{00} & m_{00} \end{array}),\theta_{p}=atan2(m_{01},m_{10})$$

(c) rBRIEF binary descriptor The sample under consideration is herein referred to as ${\left\{\Delta_{k}^{\left(1\right)},\Delta_{k}^{\left(2\right)}\right\}}_{k=1}^{L}$. Following the rotation by $\theta_{p}$, the sampling within the neighborhood results in the L-bit descriptor d(p).

(25) $$b_{k}=\left\{\begin{array}{c} 1,I(p+R(\theta_{p})\Delta_{k}^{\left(1\right)})<I(p+R(\theta_{p})\Delta_{k}^{\left(2\right)})\\ 0,otherwise \end{array}\right.,d(p)=[b_{1},\ldots ,b_{L}]\in {\left\{0,1\right\}}^{L}$$

In this context, R(θ) denotes a two-dimensional rotation matrix, frequently represented as L∈{256,512}.

#### 2.3.2. Fast Matching Based on FLANN-LSH

Efficient matching is carried out to pair descriptors across images using approximate nearest neighbor search and ratio testing.

The retrieval of binary descriptors is achieved through the utilization of the Hamming distance metric. In the presence of two descriptors, the term $a,b\in {\left\{0,1\right\}}^{L}$ is employed.

(26) $$d_{H}(a,b)=\sum_{k=1}^{L}1[a_{k}⊕b_{k}]$$

The symbol ⊕ is used to denote the exclusive OR operation. FLANN employs LSH (locally sensitive hashing) to construct indexes and returns approximate nearest neighbors. The Lowe’s ratio test is employed to suppress ambiguous matches. That is to say, if query a’s nearest neighbor $b_{1}$ and second-nearest neighbor $b_{2}$ in the database satisfy.

(27) $$\frac{d_{H}(a,b1)}{d_{H}(a,b2)}<\tau ,\tau \in [0.7,0.9]$$

In the event that the aforementioned criteria are met, it can be concluded that $\left(a,b_{1}\right)$ is a candidate match.

#### 2.3.3. RANSAC False Matching Elimination and Model Robust Estimation

Robust fitting is applied to eliminate outliers and estimate a stable affine transformation.

The following model is employed to illustrate the matching point pairs $\left(x,x^{\prime }\right)$ (in homogeneous coordinates $x={\left[\begin{array}{ccc} x & y & 1 \end{array}\right]}^{T}$) using a two-dimensional affine model.

(28) $$x^{\prime }\approx Ax+t,A\in R^{2\times 2},t\in R^{2}$$

RANSAC is a probabilistic technique that samples a minimum number of point pairs (three pairs for affine transformations) to estimate (A,t). This estimate is subsequently used to calculate the reprojection error.

(29) $$e={\left\|x^{\prime }-(Ax+t)\right\|}_{2}$$

In the context of e<ε (pixel threshold), the classification of an interior point is predicated on the iterative selection of the model that encompasses the maximum number of interior points, followed by a refinement process utilizing the least-squares objective on the interior point set.

(30) $$\underset{A,t}{\min}\sum_{(x,x^{\prime })\in I}\rho ({\left\|x^{\prime }-(Ax+t)\right\|}_{2})$$

#### 2.3.4. Affine Matrix Solution

The affine parameters are solved by least-squares estimation over inlier sets.

The interior point pairs are to be stacked into a system of linear equations, and the vector $\theta ={\left[a_{11},a_{12},t_{x},a_{21},a_{22},t_{y}\right]}^{⊤}$ is to be solved for.

(31) $$\left[\begin{array}{cccccc} x & y & 1 & 0 & 0 & 0\\ 0 & 0 & 0 & x & y & 1 \end{array}\right]\theta =\left[\begin{array}{l} x^{\prime }\\ y^{\prime } \end{array}\right]$$

The minimum-squares solution is applicable to all interior points.

#### 2.3.5. Rotation Angle Extraction

The final bolt rotation angle is derived from the affine transformation, with reliable isolation of the rotational component.

In the event that an approximation is obtained through the implementation of similarity transformations—with each transformation characterized by a homogeneous scaling of s—the resultant approximation is indicated by A≈sR, where R∈SO(2) denotes the rotation angle $\theta =atan2(a_{21},a_{11})$.

In a more general sense, the polar decomposition of an affine matrix A=RS (where S is symmetric and positive definite) should be performed.

(32) $$R=A{\left(A^{⊤}A\right)}^{-\frac{1}{2}},\theta =atan2(R_{21},R_{11})$$

The rotational component can be reliably extracted even under slight rotation. The resulting value θ∈(−π,π] is indicative of the rotational angle of the bolt.

### 2.4. Bolt Inspection and Angle Estimation

The present paper proposes an integrated bolt loosening detection process that combines object detection and angle estimation. First, leveraging the aforementioned improved object detection network, it achieves precise localization of bolt targets amidst complex backgrounds and multi-scale interference. This network employs MobileViT as its lightweight backbone, incorporating the CBAM attention mechanism before SPPF-LSKA to enhance spatial and channel feature selection capabilities. When employed in conjunction with CARAFE upsampling and BiFPN-C2f_UIB for efficient multi-scale fusion, it exhibits a capacity to maintain high detection accuracy and real-time performance even in scenarios characterized by the presence of dense small objects and low contrast.

The ORB (Optical Robustness Benchmark) algorithm is utilized for keypoint detection and descriptor generation, offering multiscale and rotation-invariance properties while balancing speed and stability. The matching phase employs FLANN (Fast Library for Approximate Nearest Neighbors) to efficiently retrieve candidate pairs, combined with RANSAC (Random Sample Consensus) to effectively eliminate mismatches and extract stable rotational components. Finally, the estimated angle is compared with the reference angle to determine bolt loosening status. This is accomplished by fitting an affine transformation using high-quality matched pairs and calculating the rotation angle. The abnormal bolts are subsequently accentuated with prominent annotations and spatially localized.

This process does not necessitate additional training, exhibits excellent real-time performance and cross-scenario generalization capabilities, and significantly enhances detection robustness and angular estimation accuracy in complex industrial environments. The system provides stable and efficient technical support for automated bolt loosening detection in structural health monitoring.

## 3. Experimental Setup

All experiments in this study were conducted in a high-performance computing environment. The experimental hardware platform comprised servers equipped with NVIDIA GeForce RTX 3090 graphics cards (24 GB VRAM) and 128 GB physical memory, powered by Intel Xeon Gold 5218 central processing units, and running Windows 10 operating systems. The PyTorch (version 2.3.0) deep learning training and inference framework was selected to fully leverage the parallel computing advantages of GPUs, ensuring the efficiency and reproducibility of large-scale experiments.

In order to systematically evaluate the adaptability and robustness of the proposed method in real-world engineering environments, experimental data were collected. The acquisition devices consisted of multiple commercial smartphones from different brands, ensuring diversity in imaging sensors and optics. The captured targets included standard petroleum flange bolts of specifications M12 and M24, together with a large number of irregular and non-standard bolts, thereby covering representative variations in size, material, and surface treatment. All images were saved in JPEG format. Given the absence of publicly available bolt inspection and defect-specific datasets, all images used in the experiment were self-collected.

The resulting dataset consists of 1200 images with approximately 3797 annotated instances (≈2601 bolts and ≈1196 nuts, reflecting the natural class distribution). To ensure broad representativeness, images were collected under diverse acquisition conditions, including different shooting distances, varied illumination (ranging from low light to high-intensity sunlight and reflective cases), and multiple viewpoints (in-plane rotations and moderate out-of-plane tilts). Furthermore, complex industrial backgrounds, partial occlusions, and surface reflections were deliberately included to mimic realistic interference factors. These acquisition strategies provide broad coverage of positional and scale variations, ensuring that the dataset is representative of real-world industrial environments.

In order to enhance the model’s generalization capability and mitigate the risks of overfitting that arise from limited sample size, this paper employs diverse data augmentation strategies during the preprocessing stage. These include enriching the original data through operations such as adding Gaussian noise, color perturbations, contrast adjustments, random rotations, horizontal/vertical flipping, and affine cropping. This series of augmentation techniques has been demonstrated to significantly increase the diversity of training samples, thereby enabling the model to better adapt to variable conditions in real-world scenarios.

The dataset was meticulously partitioned into training, validation, and test sets at ratios of 70%, 15%, and 15%, respectively. Each segment was designed to ensure balanced data distribution, thereby maintaining scientific validity and representativeness in experimental evaluations. The model’s performance was then systematically evaluated and compared across a range of environments, lighting conditions, and bolt states. This comprehensive approach enabled the verification of the proposed method’s practicality and robustness in complex industrial settings.

## 4. Results

### 4.1. Performance Indicators

In order to provide a comprehensive evaluation of the effectiveness of bolt target detection and angle estimation methods, this paper employs multiple mainstream quantitative metrics, including Accuracy, Precision, Recall, F1 Score, Average Precision (AP), and Mean Average Precision (*mAP*). These metrics objectively reflect the model’s overall performance across different task dimensions.

In the context of the bolt detection task, the model generates predictions for each input image. Subsequently, the model’s performance must be evaluated by comparing the predictions to manually annotated ground truth values. A taxonomic classification of the samples is possible on the basis of the correspondence between predicted results and true categories. The samples can be categorized into four classes.

The term “true positive” (*TP*) refers to a positive test result that is subsequently confirmed as accurate. The quantity of samples that are determined to be bolts and are accurately identified by the model.

A false positive (*FP*) is defined as the number of samples incorrectly identified as bolts by the model, but which are, in fact, non-bolts.

A false negative (*FN*) is defined as a result that is negative but should be positive. The quantity of instances in which the model failed to identify bolts as such, despite their actual presence.

The term “true negative” (*TN*) is used to denote a specific category of results in diagnostic tests. The quantity of samples that were determined to be non-bolts and not identified by the model is indicated herein.

In light of the aforementioned statistics, the definitions of pertinent evaluation metrics are as follows:

(1) *Recall*

The recall rate is indicative of the model’s capacity to detect all genuine bolt targets.

(33) $$Recall=\frac{TP}{TP+TN}$$

(2) *Precision*

Precision is defined as the proportion of predictions classified as bolts that are, in fact, bolts.

(34) $$Precision=\frac{TP}{TP+FP}$$

(3) Average precision

The Average Precision metric is a quantitative assessment of a model’s capacity to detect a specific target category with an average precision rate. The next step is to plot a precision–recall curve with recall on the *x*-axis and precision on the *y*-axis. The area under this curve is referred to as *AP*.

(35) $$AP=\int_{0}^{1}P(r)dr$$

In this context, *P* represents precision, and *r* represents recall.

(4) Mean average precision

*mAP* is a metric that reflects the average detection accuracy of a model across all detection categories. It is considered a core metric for evaluating the performance of object detection algorithms. The term is defined as the arithmetic mean of the *AP* values for each category.

(36) $$mAP=\frac{1}{N}\sum_{1}^{N}AP_{i}$$

In this context, *N* denotes the total number of detection categories, while $AP_{i}$ represents the average precision for the i-th target category.

### 4.2. Improved YOLOv8 Network Experiment Results

The enhanced YOLOv8 model (hereinafter referred to as Ours-Det) proposed in this study exhibits substantial advantages in bolt detection tasks, achieving higher detection accuracy and robustness, particularly in complex environments and scenarios characterized by dense small objects. A thorough examination of the performance metrics reveals that, on the validation set, Ours-Det attains a mAP@0.5 of 96.0%, mAP@0.5:0.95 of 81.2%, Precision (P) of 96.3%, and Recall (R) of 88.0%. A comparison of the mAP@0.5:0.95 metric with the baseline YOLOv8 model, trained using identical strategies, reveals significant enhancements, particularly in the detection of small objects and in low-contrast scenarios. The mAP@0.5:0.95 model demonstrates notable improvements in detection stability and localization accuracy. The findings indicate that the proposed structural enhancements effectively improve the model’s detection performance under complex working conditions.

**Figure 10:**
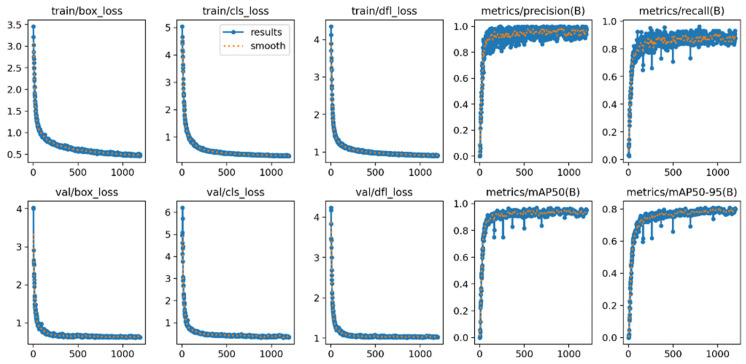
Training results.

During the training process, the loss functions associated with localization, classification, and regression all undergo a rapid decline, converging within a limited number of epochs. The overall curves demonstrate smooth trajectories without oscillations or indications of overfitting, thereby substantiating the model’s robust fitting capability and stability (see Figure 10). Subsequent observation of training curves under learning rate warm-up and Cosine annealing strategies indicates that the mAP metric attains a stable plateau phase after approximately 200 iterations, suggesting sustained and consistent learning performance during protracted training.

**Figure 11:**
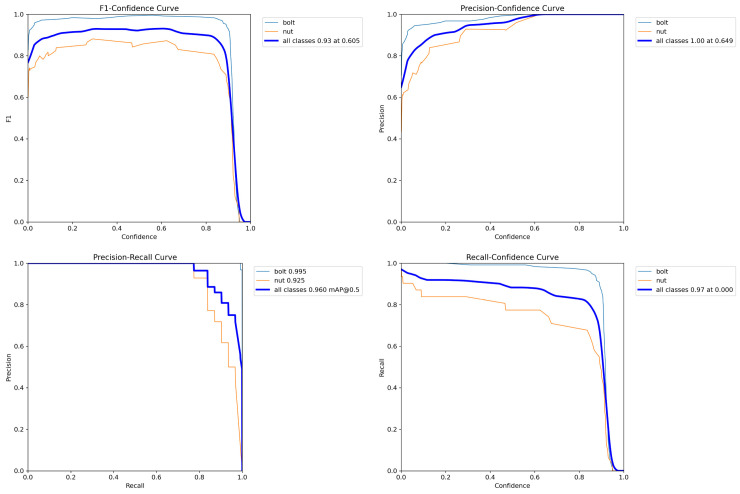
Performance diagnostic curves of F1-confidence, precision–confidence, precision–recall, and recall-confidence.

With regard to the performance visualization diagnostics (see Figure 11), the F1-Confidence, P-Confidence, P-R, and R-Confidence curves demonstrate reasonable trends. The F1 curve attains its optimal operating point at approximately 0.60 confidence threshold, where the maximum F1 value is approximately 0.93. This indicates that the model achieves a high balance between precision and recall. The Precision-Confidence curve demonstrates that the model maintains exceptionally high precision (close to 1.0) as confidence increases. In contrast, the Recall–Confidence curve demonstrates a modest decrease in recall within the high-confidence region, a phenomenon that is closely associated with nut occlusion and reflective interference, which are prevalent in industrial contexts. The model demonstrates a high degree of precision while exhibiting a commendable recall performance, accompanied by a substantial reduction in false positive rates.

**Figure 12:**
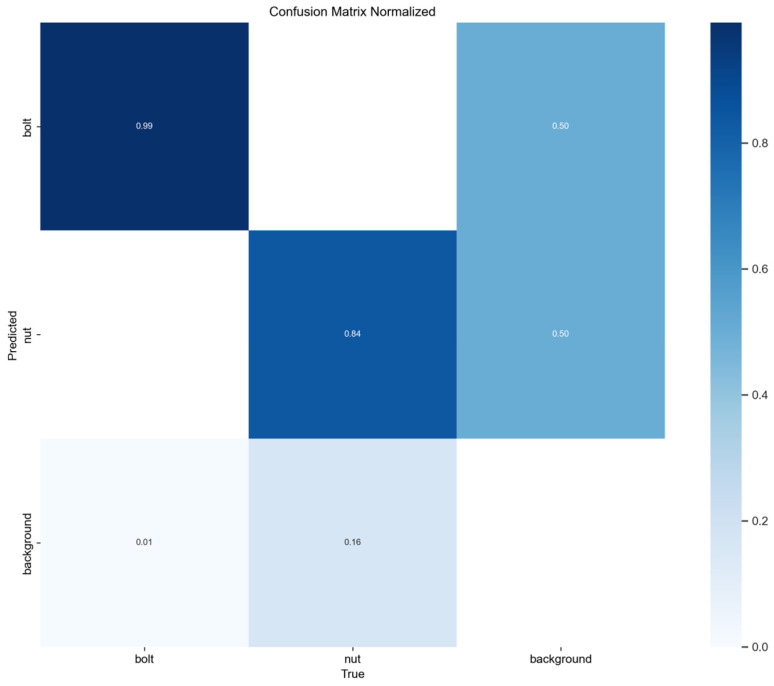
Confusion matrix of the improved YOLOv8 model.

The results of the confusion matrix are displayed in Figure 12. The detection accuracy and recall for the “bolt” category approach saturation (detection accuracy ~99%), with virtually no missed detections. The accuracy of the “nut” category ranges from approximately 84% to 93%, with misclassifications primarily stemming from light reflections and partial occlusions, where some samples are mistakenly classified as background. This phenomenon closely aligns with real-world industrial scenarios and highlights the challenges in distinguishing similar objects against complex backgrounds. Reverse analysis of error samples indicates that CARAFE’s content-aware feature reorganization mechanism holds potential for improving nut edge feature recovery, while BiFPN + C2f_UIB’s multi-scale fusion capability effectively enhances cross-scale consistency modeling, providing structural support for robust detection in complex scenarios.

In summary, experimental results conclusively demonstrate that Ours-Det outperforms both native YOLOv8 and comparison models in overall accuracy and robustness, exhibiting notably higher detection stability in scenarios with dense small objects and complex backgrounds. The model’s efficacy is attributed to its capacity for rapid convergence, minimal false detection rates, and robust generalization, achieved through the integration of feature enhancement mechanisms such as CBAM and SPPF-LSKA, complemented by multi-scale fusion designs including CARAFE and BiFPN + C2f_UIB. This provides reliable technical assurance for practical applications in bolt loosening detection.

### 4.3. Baseline Experiment Results

To further validate the comprehensive performance of the enhanced model Ours-Det, a systematic comparison was conducted with current mainstream detectors, including Faster R-CNN, SSD, YOLOv5, YOLOv6, and the native YOLOv8. For fairness, all baselines were evaluated on the same dataset using their large/high-capacity variants (as reflected in the parameter counts of Table 1), with 640 × 640 input resolution, and a unified training protocol (SGD with cosine learning schedule, batch size 16, up to 2000 iterations with early stopping once validation performance plateaued). The comparison metrics included detection accuracy (P, R, mAP@0.5, mAP@0.5:0.95) and model scale (parameter count and storage size), providing a balanced assessment of both accuracy and efficiency for practical engineering applications.

The experimental results are presented in Table 1. Ours-Det model exhibits a substantial enhancement in detection accuracy, with mAP@0.5 reaching 96.0% and mAP@0.5:0.95 reaching 81.2%, representing improvements of approximately +2.0 and +5.8 percentage points over the original YOLOv8 model, respectively. This enhancement is particularly evident under stringent IoU thresholds, indicative of the enhanced network’s capacity to model intricate boundaries and geometric consistency in challenging scenarios, such as complex backgrounds, small objects, and partial occlusions. It is noteworthy that the precision and recall metrics attained 96.3% and 88.0%, respectively, thereby achieving high recall while maintaining low false positive rates. This outcome is indicative of the model’s capacity to strike an optimal balance between accuracy and comprehensiveness.

In terms of model size, Ours-Det features 247,953 parameters (approximately 2.5 million) and occupies 5.6 MB of storage—significantly lower than YOLOv8’s 43.6 million parameters and 87.7 MB, and substantially outperforming other mainstream detectors. This lightweight design substantially reduces computational and storage overhead, thereby enabling deployment feasibility on resource-constrained embedded platforms and edge computing devices. Notwithstanding constrained hardware, it guarantees real-time and reliable execution of detection operations.

In summary, Ours-Det demonstrates superiority over existing representative methods in two key areas: detection accuracy and lightweight design. This finding signifies noteworthy engineering adaptability and practical value.

### 4.4. Modular Ablation Experiments

In order to thoroughly investigate the contribution of each improvement module to overall performance, this study conducted systematic ablation tests under unified experimental conditions. The MobileViT backbone (MV), the SPPF-LSKA module (SL), the CBAM attention mechanism (CB), the CARAFE upsampling module (CA), and the BiFPN + C2f_UIB fusion structure (BF) were progressively introduced. Subsequently, a comparative analysis of detection metrics was conducted across various combinations within the validation set.

The experimental results are presented in Table 2. It has been demonstrated that when utilizing solely the MobileViT backbone, the model attains performance that is comparable to mAP@0.5 = 0.950 and mAP@0.5:0.95 = 0.763. This observation signifies that the lightweight MobileViT backbone is well-suited for efficiently extracting both local and global features. The incorporation of the SPPF-LSKA module into the model has been shown to result in a significant enhancement, with a 1.7-point increase in mAP@0.5:0.95, as measured by the strict metric. This improvement underscores the module’s efficacy in modeling long-range dependencies and suppressing complex backgrounds, thereby demonstrating its potential for advanced analysis and interpretation. Subsequent introduction of the CBAM attention mechanism led to a 1.3-point increase in mAP@0.5:0.95, accompanied by improvements in both precision and recall. These findings suggest that spatio-channel joint attention contributes to the effective highlighting of key regions and the suppression of redundant features. The incorporation of the CARAFE upsampling module led to a further optimization of edge recovery and small-object detail extraction, resulting in an increase in mAP@0.5:0.95 to 0.804. This outcome demonstrates the efficacy of the content-aware feature reconstruction mechanism in modeling complex structural boundaries. The integration of the BiFPN + C2f_UIB module has been demonstrated to enhance consistency across multi-layer features. This enhancement is attributed to the module’s capacity for effective cross-scale information aggregation and bottleneck compression mechanisms. This culminated in optimal performance on the validation set, with mAP@0.5 = 0.960 and mAP@0.5:0.95 = 0.812.

The findings indicate that each module contributes independently to performance gains and exhibits significant complementary effects through synergistic interaction. The simultaneous introduction of SPPF-LSKA and CBAM has been shown to enhance long-range dependencies and discriminative feature selection, particularly improving recognition of small and low-contrast objects. CARAFE’s content-aware feature rearrangement effectively restores edge and texture details, while BiFPN + C2f_UIB’s multi-scale fusion ensures consistency and integrity of cross-resolution features. Sensitivity analysis further demonstrates that Ours-Det achieves significant improvements over native YOLOv8 in small object AP, aligning closely with the aforementioned modules’ mechanisms for detail restoration and cross-scale modeling.

The efficacy and complementarity of each proposed enhancement module is substantiated by the results of the ablation experiments. MobileViT provides a lightweight, high-quality feature representation foundation for the entire network. SPPF-LSKA and CBAM have been shown to enhance both global and local features, while CARAFE has been demonstrated to excel in edge detail recovery. BiFPN + C2f_UIB has been demonstrated to achieve efficient and robust cross-scale fusion. In essence, the synergistic interaction of these modules facilitates Ours-Det’s capacity to achieve high-precision detection of bolt targets in complex scenarios, while concurrently maintaining a lightweight and stable performance.

### 4.5. Experiment on Bolt Loosening Angle Detection Accuracy Under Standard Conditions

The objective of this experiment is to systematically evaluate the detection accuracy and stability of the proposed algorithm for bolt rotation angles under standardized conditions. To guarantee the diversity of test samples and the scientific rigor of the evaluation, this section selects two representative real-world bolt images for testing. These images exhibit significant differences in background texture, lighting conditions, reflectivity, and surface texture complexity. This enables the model to demonstrate its adaptability to various interference factors within a limited sample set. The actual rotation angles of all test samples are known and serve as the evaluation benchmark.

The following experimental comparison methods are employed:

The proposed method is as follows: ORB incorporates features that facilitate effective matching through FLANN and robust estimation through RANSAC.

The first control group is composed of SIFT features in conjunction with RANSAC matching.

The second control group is characterized by the implementation of a hough circle detection method. This approach involves the extraction of rotation angles based on shape parameters.

All samples underwent YOLOv8 detection and ROI extraction, followed by sequential application of the algorithm’s multi-stage feature extraction, matching, and angle recognition process to output rotation angle detection results. The detection errors for each sample were statistically calculated relative to the target angle. The evaluation metrics employed include the mean absolute error (MAE), the maximum error (MaxE), the standard deviation (Std), and the accuracy (Acc). Accuracy is defined as the proportion of samples where the detected angle corresponds to the true angle within the tolerance range of ±1°.

**Figure 13:**
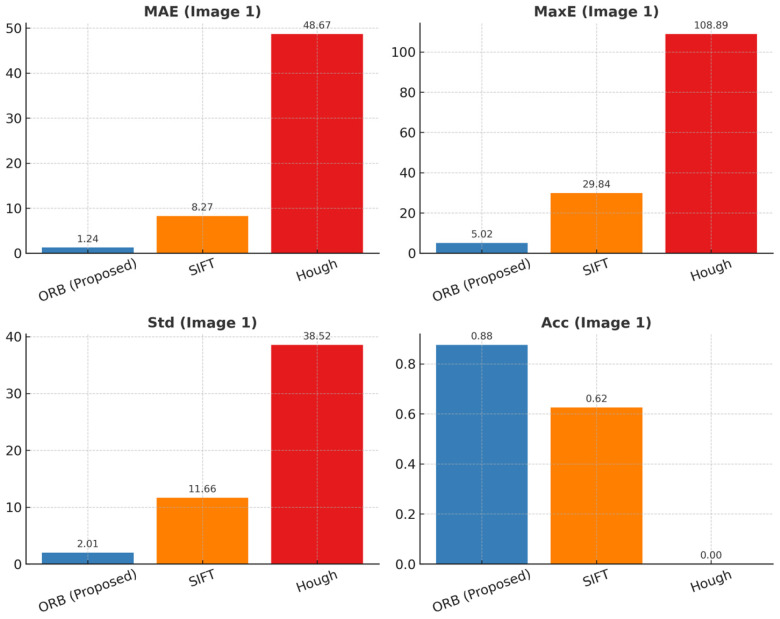
Results of angle calculation evaluation indicators in image 1.

As shown in Figure 13 (corresponding to the raw values in Table 3), the proposed ORB method attains the lowest error (MAE 1.24°, MaxE 5.02°) and the highest accuracy (Acc 0.88) on Image 1, while SIFT shows moderate errors and Hough fails under illumination/reflection, leading to large MaxE and Std.

**Figure 14:**
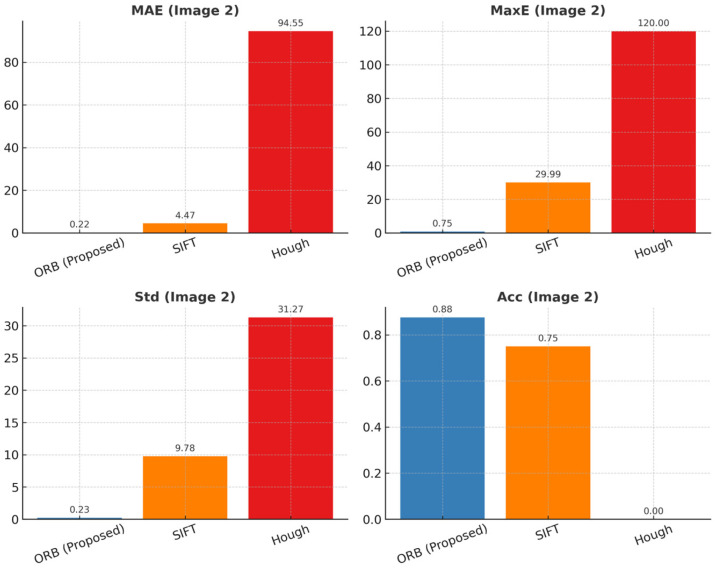
Results of angle calculation evaluation indicators in image 2.

Table 4 summarizes the results of image 2, with corresponding indicators shown in Figure 14. Again, ORB maintains stable accuracy (Acc = 0.88) with low error variance, whereas SIFT exhibits moderate performance and Hough fails under complex illumination and reflections.

**Figure 15:**
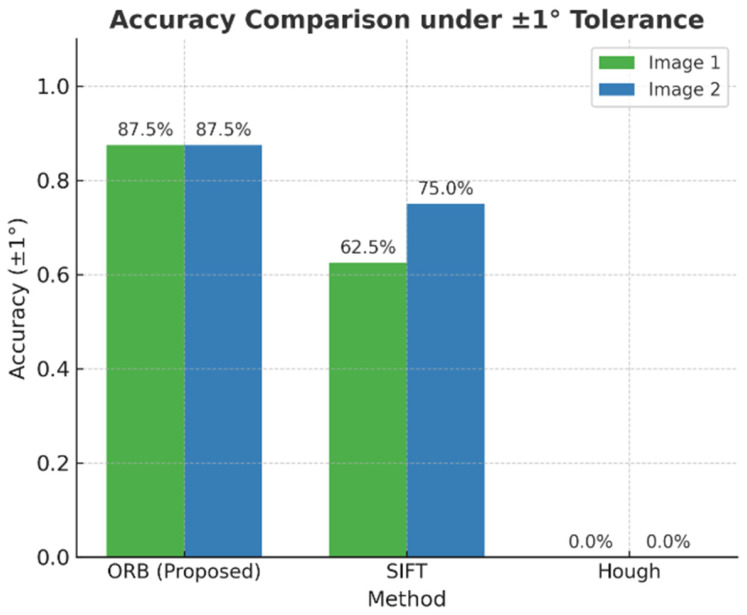
Comparison of angle calculation accuracy.

Figure 15 compares overall accuracy under the ±1° tolerance across both images. ORB achieves 87.5% accuracy in both cases, outperforming SIFT (62.5–75%) and Hough (0%).

The experimental results are displayed in the figure. It has been demonstrated that under standardized conditions, the proposed method (ORB, Proposed) exhibits excellent angle detection performance in both test images. With regard to accuracy, ORB attained 87.5% in both experimental sets, thereby demonstrating a substantial improvement over SIFT (62.5–75%) and Hough (0%). This outcome indicates the capacity of ORB to function reliably under conditions involving intricate backgrounds and lighting variations. With respect to error control, ORB achieves a mean absolute error (MAE) of 1.24° (Image 1) and 0.22° (Image 2) and a maximum error (MaxE) of only 5.02° (Image 1) and 0.75° (Image 2), which is substantially lower than the values obtained with SIFT (MAE 8.27°/4.47°, MaxE 29.84°/29.99°) and Hough (MAE 48.67°/94.55°, MaxE 108.89°/170.00°). With regard to variability, ORB’s standard deviation (Std) is 2.01° (Image 1) and 0.23° (Image 2), which is significantly higher than the 10.72° standard deviation of SIFT and the 34.90° standard deviation of Hough. This indicates that ORB produces smoother outputs under conditions involving reflective lighting and low-texture. The proposed method demonstrates an accuracy of 87.5%, with a ±1° tolerance, while traditional methods exhibit unstable performance or complete failure due to limitations such as local feature drift or geometric fitting errors.

The underlying factors contributing to these disparities in performance are as follows: SIFT is susceptible to mismatches in high-frequency texture regions, with descriptors demonstrating excessive sensitivity to repeated edges and local noise. Hough circle detection is predicated on the ideal circularity assumption, resulting in substantial fitting deviations under reflective and shadowed conditions, with deviations reaching hundreds of degrees. Conversely, ORB employs a strategy that facilitates efficient keypoint extraction through the utilization of lightweight FAST detection and BRIEF descriptors. When employed in conjunction with FLANN matching and RANSAC robust fitting, it effectively eliminates anomalous matches and ensures stable rotation angle estimation.

In summary, experimental results clearly validate the comprehensive advantages of the proposed method in achieving high accuracy, strong robustness, and low computational overhead, making it suitable for bolt loosening detection tasks in industrial settings.

### 4.6. Comprehensive Performance Evaluation Experiment

**Figure 16:**
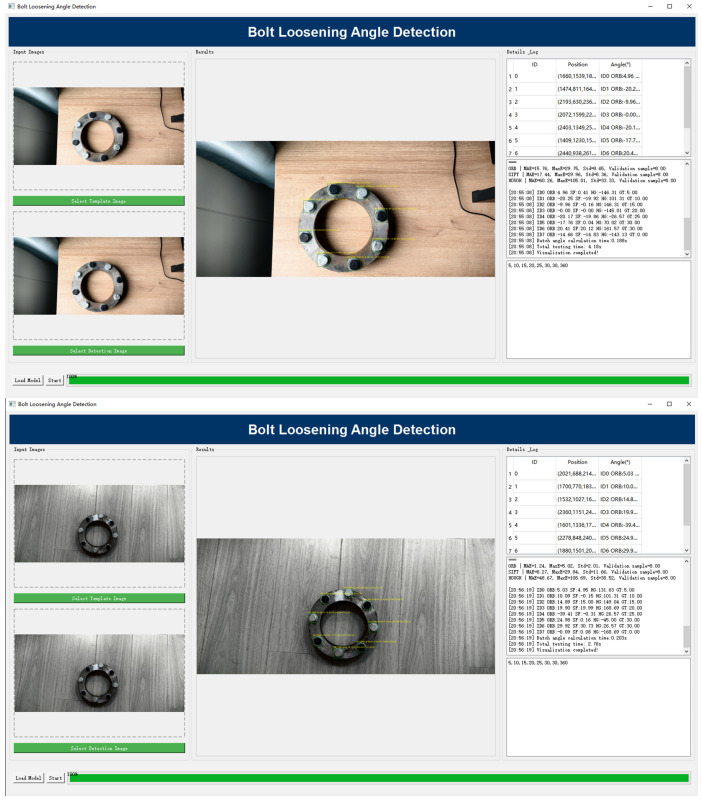
Software interface in two typical application scenarios (shown vertically). **Top**: daylight/color scene; **Bottom**: grayscale/low-texture scene. **Left**—input thumbnails; **Center**—detection view with bolt keypoints and angle overlays; **Right**—per-bolt results list (ID, position, angle) and processing log.

In order to provide a comprehensive evaluation of the applicability and stability of the constructed bolt loosening detection system in actual engineering scenarios, this experiment assessed its overall performance under multiple complex operating conditions. The system integrates two core functions: bolt target detection and rotation angle estimation. The software interface facilitates the real-time presentation of detection results and status assessments, thereby ensuring efficiency and operability throughout the entire process, from data acquisition to result output. Figure 16 illustrates two representative application scenarios presented vertically: the top screenshot shows a daylight/color case, and the bottom screenshot shows a grayscale/low-texture case. The interface comprises an input thumbnail panel (left), a detection/result view with keypoints and angle overlays (center), and a results list plus processing log (right), enabling users to track per-bolt IDs, positions, and estimated angles in real time.

During the testing phase, the system was subjected to a range of lighting conditions, viewing angles, and background interference. These conditions were designed to assess the system’s capability to localize bolts in real-time and perform angular measurement with precision. Concurrently, the interface exhibited the detection locations, the classification information, and the results of the rotation angle determination. As demonstrated in Figure 16, the interface intuitively presents the spatial distribution of detection targets and their loosening status.

The evaluation results demonstrate that the system maintains stable detection accuracy and angle estimation capabilities even in complex environments, while processing speeds meet real-time requirements. In practical testing, the system rapidly completes target recognition and angle output under conditions such as intense lighting variations, complex background textures, or deviating shooting angles, exhibiting excellent robustness and environmental adaptability. This performance is the result of an effective collaboration between detection and matching modules. Front-end target detection precisely locates bolt regions, providing reliable input for subsequent angle calculations. Rear-end angle estimation, supported by multi-scale features and optimized matching strategies, effectively mitigates adverse factors such as lighting interference, weak textures, and local reflections.

A thorough examination has been conducted, and the results indicate that the system demonstrates high levels of detection accuracy, angle estimation reliability, processing efficiency, and adaptability to complex operating conditions. These findings validate the system’s feasibility and stability for practical engineering applications. This achievement provides a viable technical solution for bolt loosening detection and lays a solid foundation for subsequent applications in operational condition monitoring of large-scale equipment and structural safety assessment.

## 5. Conclusions and Discussion

In order to address the challenges of missed detections, insufficient feature representation, and inadequate cross-scale information fusion in complex backgrounds, weak textures, and densely distributed scenarios for flange bolts, this paper systematically enhances the lightweight and robustness of both the YOLOv8 detection and angle estimation pipelines, achieving closed-loop implementation at the system level. In the context of detection, MobileViT substitutes conventional, cumbersome convolutional backbones. It accomplishes this by concurrently capturing local textures and global context with a minimal number of parameters. Separable Large-Kernel Convolutional Attention (LSKA) is embedded within SPPF, while Convolutional Block Attention Module (CBAM) is introduced in its preprocessing stage to achieve joint spatial–channel modeling and salient region enhancement. The upsampling stage employs CARAFE for content-aware reconstruction, with the objective of maximizing high-resolution detail preservation. The enhancement of neck structure employs BiFPN and an advanced C2f_UIB, with the objective of reinforcing bidirectional contextual flow and cross-scale fusion. These modifications have been shown to enhance detectability and localization accuracy for small, low-contrast targets without compromising inference speed, thereby meeting the real-time deployment demands characteristic of edge and embedded scenarios.

The angle estimation module eschews reliance on specific markers and complex descriptors, establishing a lightweight, robust workflow based on ORB + FLANN + RANSAC. ORB extracts multi-scale rotation-invariant keypoints and binary descriptors, combined with FLANN for efficient approximate matching. Within the RANSAC fitting framework, anomalous matching points are discarded to extract stable rotation components. This pipeline circumvents the need for manual annotation or the utilization of complex operators, thereby ensuring the maintenance of high in-point coverage and directional consistency under conditions of strong reflections, mild blurring, and moderate viewpoint deviations. This effectively controls variance fluctuations in angle estimation, ensuring real-time performance and stability on low-computational-power platforms.

Quantitative outcomes under standardized tests corroborate the above: with a ±1° tolerance, accuracy reaches 87.5% on both test images; the mean absolute error (MAE) is 1.24° and 0.22°, and the maximum error (MaxE) is 5.02° and 0.75°, respectively—consistently outperforming SIFT and Hough baselines. Coupled with a runnable software interface that visualizes detections and per-bolt angles in real time, the system satisfies the intended objectives of robustness and deployability.

In essence, the multi-level modifications outlined in this paper serve to enhance the detection branch’s capacity to identify small targets and process cross-scale information flows. The angle branch plays a pivotal role in ensuring the consistency of quality by leveraging ORB’s efficient keypoint detection and RANSAC robust fitting methodologies. When integrated at the system level, these components achieve lightweight, accurate, and real-time detection of flange bolt loosening. Therefore, the study achieves its stated targets of lightweight design, accurate and real-time bolt loosening detection, and practical software realization for edge/industrial terminals. Subsequent research will concentrate on three domains: First, there is a necessity to enhance data acquisition and adaptive thresholding strategies for extreme conditions to improve robustness under strong reflections and low-light scenarios. Second, there is a need to introduce self-supervised/domain adaptation without altering the existing lightweight framework to reduce cross-scenario transfer costs. Third, there is a need to extend to multi-bolt coordination and temporal consistency modeling to establish continuous diagnostic capabilities from single-frame detection to online health monitoring. In addition, future work will address more challenging failure cases such as heavy occlusion, strong reflections, and bolt surface corrosion, for which multimodal sensing strategies may provide effective solutions. These directions will further solidify the practical value and scalability of this method in industrial environments with limited resources.

## Figures and Tables

**Table 1:** Experimental results of the baseline model.

Model	P	R	mAP@0.5	mAP@0.5:0.95	Parameter (M)	Model Size (MB)
Faster R-CNN	0.880	0.861	0.900	0.752	60.2	234
SSD	0.870	0.842	0.900	0.755	23.3	90
YOLOv5	0.932	0.905	0.939	0.746	53.1	106.8
YOLOv6	0.866	0.892	0.899	0.721	110.9	222.3
YOLOv8	0.942	0.904	0.940	0.754	43.6	87.7
Ours-Det	0.963	0.880	0.960	0.812	2.48	5.6

**Table 2:** Results of ablation experiments.

MV	SL	CB	CA	BF	P	R	mAP@0.5	mAP@0.5:0.95
1	0	0	0	0	0.931	0.870	0.950	0.763
1	1	0	0	0	0.942	0.876	0.954	0.780
1	1	1	0	0	0.951	0.880	0.957	0.793
1	1	1	1	0	0.960	0.885	0.960	0.804
1	1	1	1	1	0.963	0.880	0.960	0.812

**Table 3:** Angle calculation results of image 1.

	ORB	SIFT	Hough	GT
ID0	5.03	4.95	131.63	5
ID1	10.09	−0.15	101.31	10
ID2	14.89	15.00	149.04	15
ID3	19.90	19.99	168.69	20
ID4	−39.41	−0.31	26.57	25
ID5	24.98	0.16	−45.00	30
ID6	29.92	30.73	26.57	30
ID7	−0.09	0.08	−18.69	360

**Table 4:** Angle calculation results of image 2.

	ORB	SIFT	Hough	GT
ID0	5.05	0.03	158.75	5
ID1	10.19	10.24	−145.01	10
ID2	14.98	15.05	165.96	15
ID3	19.65	20.07	63.43	20
ID4	24.97	25.18	−147.99	25
ID5	30.30	29.73	−159.44	30
ID6	29.25	0.01	−153.43	30
ID7	−0.06	−0.03	−180.00	360

## Data Availability

Data will be made available on request.
